# Flow Cytometric Quantification of HIV-1-Infected Cells Expressing Either Abortive or Elongated HIV-1 Transcripts Using Flow-FISH

**DOI:** 10.21769/BioProtoc.5392

**Published:** 2025-07-20

**Authors:** Shirley Man, Teunis B.H. Geijtenbeek, Neeltje A. Kootstra

**Affiliations:** 1 Department of Experimental Immunology, Amsterdam UMC Location University of Amsterdam, Amsterdam, Netherlands; 2 Amsterdam Institute for Immunology and Infectious Diseases, Amsterdam, Netherlands

**Keywords:** HIV-1 reservoir, HIV-1 latency, Abortive HIV-1 TAR RNA, Flow-FISH, Flow cytometry, Reservoir detection

## Abstract

The persistence of the HIV-1 reservoir remains the ultimate obstacle in achieving a cure. Cure strategies targeting the HIV-1 reservoir are under development, and therefore, finding ways to improve the detection of the reservoir is crucial. Several reservoir detection techniques exist to assess different markers of the HIV-1 reservoir, such as PCR-based assays and protein-based flow cytometric methods. We developed a flow cytometry-fluorescent in situ hybridization (flow-FISH) approach that assesses HIV-1 at the transcriptional level. Using a combination of probes that target either the HIV-1 trans-activation response (TAR) region and 5′ long terminal repeat (LTR) or the Gag sequence, our assay distinguishes between infected cells expressing abortive or elongated HIV-1 RNAs. This assay utilizes the branched-DNA method to amplify the fluorescent signal of the hybridized RNA probes and can be used directly for thawed or cultured cells, with the option to include surface antibody staining. Cellular expression of abortive and/or Gag HIV-1 RNAs is measured by flow cytometry. Our flow-FISH approach gives insight into the transcriptional dynamics of the HIV-1 reservoir and allows for the characterization of latently infected cells.

Key features

• Detection of latently HIV-1-infected cells identified by the expression of abortive HIV-1 TAR transcripts.

• Cell activation is not required for HIV-1 detection; therefore, the cellular phenotypic landscape remains preserved.

• Can be used for direct ex vivo measurements in isolated cells, such as PBMCs, from untreated and antiretroviral therapy (ART)-treated people with HIV-1 (PWH).

## Graphical overview


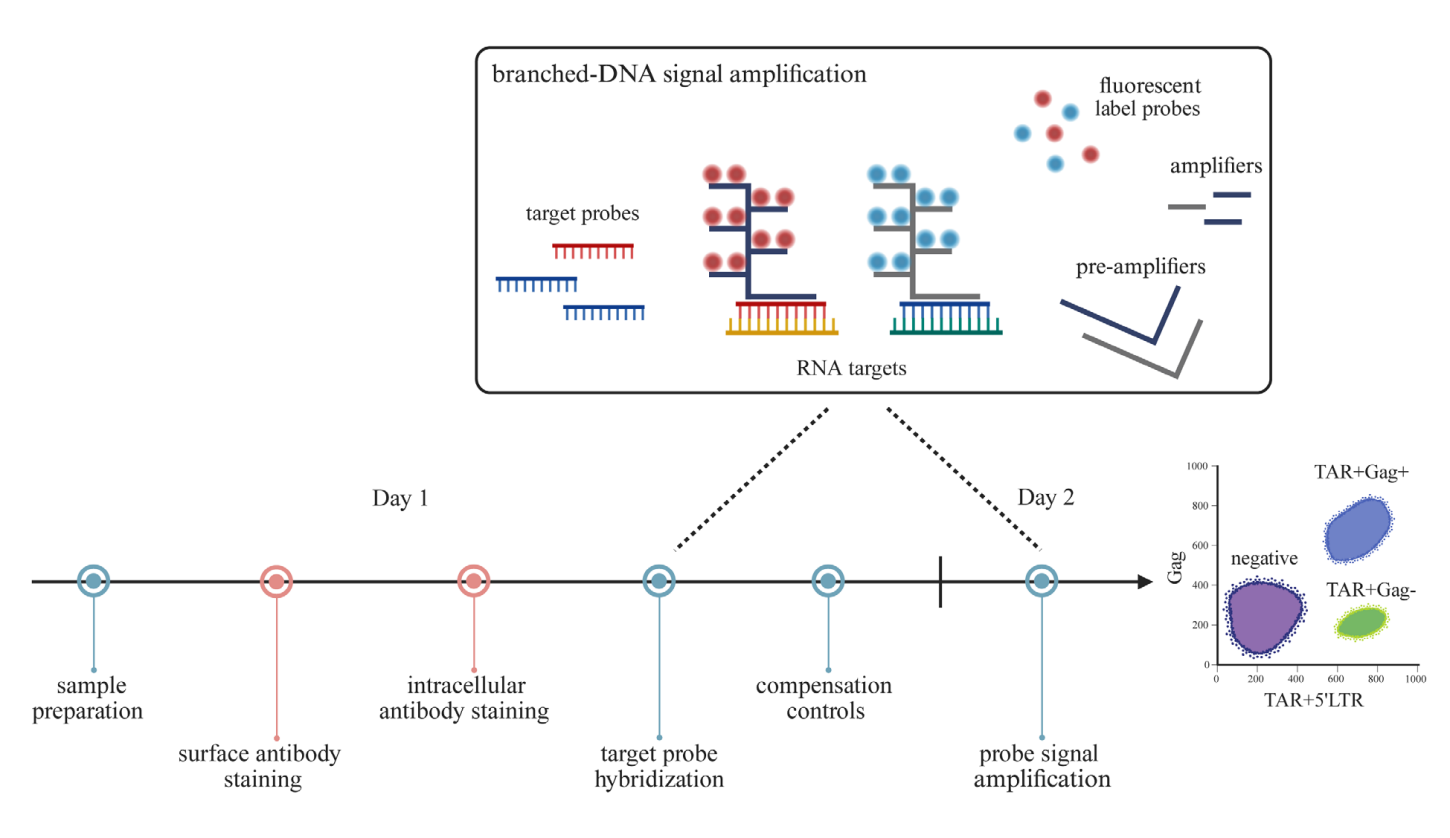


## Background

Detecting and monitoring the HIV-1 reservoir of people with HIV-1 (PWH) is crucial in advancing cure strategies. Various detection methods quantify the viral reservoir based on the different characteristics of the provirus (i.e., defect, intact, transcriptionally active, translationally active, or replication-competent provirus) [1–6]. The lack of viral antigen expression associated with HIV-1 latency complicates the assessment of the viral reservoir at the cellular level. To address this problem, we developed an assay capable of detecting these latently infected cells by targeting abortive HIV-1 RNA. Inefficient transcriptional elongation of the HIV-1 genome can lead to the production of short abortive transcripts that comprise the TAR region—a highly conserved RNA sequence found at the 5′ end of HIV-1 transcripts. These abortive TAR transcripts have been identified in latency cell lines and in resting CD4^+^ T cells from antiretroviral therapy (ART)-treated PWH [7–9]. We thus designed a flow cytometry–fluorescent in situ hybridization (flow-FISH) assay, in which flow cytometry is combined with RNA-binding probes and branched-DNA technology to amplify the fluorescent signal. We designed one probe that targets the trans-activation response (TAR) region to detect all HIV-1 RNAs, including abortive transcripts, and another probe that targets the Gag region for the detection of elongated HIV-1 RNAs. These probes allow for flow cytometric quantification of HIV-1-infected cells expressing only abortive TAR RNAs or elongated HIV-1 RNAs—a proxy for active HIV-1 transcription. In addition, we designed a third probe that binds the 5′ long terminal repeat (5′LTR; R-U5)—a sequence directly downstream of TAR but upstream of Gag—to boost the TAR probe signal for abortive transcripts with a block to elongation. It is important to note that these probe combinations do not differentiate between cells containing replication-competent and defective proviruses. Nevertheless, our flow-FISH assay is a useful tool in monitoring the HIV reservoir and can be used to phenotypically characterize latently infected cells by flow cytometry. While this protocol is optimized for peripheral blood mononuclear cells (PBMCs), this assay can also be adapted for the assessment of the viral reservoir within lymphoid tissues of PWH.

## Materials and reagents

**Biological materials**

1. ACH-2, a latently HIV-1-infected T-cell line derived from CEM cells (NIH AIDS Reagent Program; RRID: CVCL_0138)

2. PBMCs from PWH and an HIV-negative blood donor; isolated fresh or frozen

**Reagents**

1. Target probe sets, 20× (custom-made, Affymetrix, Santa Clara, CA, USA; see Table S1 for used target sequences from HIV-1 clade B); store at -20 °C

a. HIV-1 TAR in Alexa Fluor 647

b. HIV-1 5′LTR in Alexa Fluor 647

c. HIV-1 Gag in Alexa Fluor 488

2. Primeflow RNA Assay kit (Invitrogen, catalog number: 88-18005), containing:

a. RNA tubes (1.5-mL microcentrifuge tubes) (Invitrogen, catalog number: 19197)

b. Fixation buffer 1A (Invitrogen, catalog number: 00-18100); store at 2–8 °C

c. Fixation buffer 1B (Invitrogen, catalog number: 00-18200); store at 2–8 °C

d. Fixation buffer 2 (8×) (Invitrogen, catalog number: 00-18400); store at 2–8 °C

e. Permeabilization buffer (10×) (Invitrogen, catalog number: 00-18300); store at 2–8 °C

f. RNase inhibitor (100×) (Invitrogen, catalog number: 00-16002); store at 2–8 °C

g. Wash buffer (Invitrogen, catalog number: 00-19180); store at 2–8 °C

h. Target probe diluent (Invitrogen, catalog number: 00-19185); store at 2–8 °C

i. PreAmp mix (Invitrogen, catalog number: 00-16000); store at 2–8 °C

j. Amp mix (Invitrogen, catalog number: 00-16001); store at 2–8 °C

k. Label probe diluent (Invitrogen, catalog number: 00-19183); store at 2–8 °C

l. Label probes (100×) (Invitrogen, catalog number: 00-16003); store at -20°C

m. Intracellular (IC) fixation buffer (Invitrogen, catalog number: 00-8222); store at 2–8 °C

n. Storage buffer (Invitrogen, catalog number: 00-19178); store at 2–8 °C

o. Compensation kit (Invitrogen, catalog number: 88-17009)

3. UltraComp eBeads^TM^ microspheres (Thermo Fisher Scientific, Invitrogen, catalog number: 01-2222); store at 2–8 °C

4. PrimeFlow^TM^ Compensation Control Alexa Fluor^TM^ 647 (Invitrogen, catalog number: PF51-17002); store at 2–8 °C

5. PrimeFlow^TM^ Compensation Control Alexa Fluor^TM^ 488 (Invitrogen, catalog number: PF51-17003); store at 2–8 °C

6. Primeflow microRNA pretreatment buffer (Invitrogen)

a. Pretreatment concentrate (4×) (Invitrogen, catalog number: 00-16009); store at 2–8 °C

b. Pretreatment diluent (Invitrogen, catalog number: 00-16008); store at 2–8 °C

7. CD4-PE antibody (RPA-T4 clone) (BioLegend, catalog number: 300507); store at 2–8 °C

8. TNF-α (PeproTech, catalog number: 300-01A); store at -20 °C

9. Phosphate-buffered saline (PBS), 1×, pH ~7.4 (Thermo Fisher Scientific, catalog number: 10010023); store at room temperature

10. Bovine serum albumin (BSA) (Merck Sigma-Aldrich, catalog number: A7906); store at room temperature

11. Ethylenediaminetetraacetic acid (EDTA) (Thermo Fisher Scientific, catalog number: 17892); store at room temperature

12. Sodium azide (Merck, Sigma-Aldrich, catalog number: S2002); store at room temperature

**Solutions**

1. FACS buffer (see Recipes)

**Recipes**

**1. FACS buffer (store at 4 °C)**

**Table:** 

Reagent	Final concentration
PBS	
BSA	0.3%
EDTA	2 mM
Sodium azide	0.01%

## Equipment

1. FACSCanto II device with lasers 488 nm, 561 nm, and 633 nm (BD Biosciences, Franklin Lakes, NJ, USA)

2. Centrifuge (ROTANTA 460R, Hettich, Kirchlengern, Germany); swinging bucket with adaptors for 15 mL conical tubes; acceleration 9, deceleration 7

3. Refrigerated centrifuge for 1.5 mL Eppendorf tubes (Eppendorf, model: Centrifuge 5430 R)

4. Heat block for 1.5 mL Eppendorf tubes (QB Series Dry Block Heating Systems, Grant Instruments, Cambridge, England)

5. Vortex mixer (Reax top, Heidolph Scientific Products, Schwabach, Germany)

## Software and datasets

1. FlowJo software version 10; application for flow cytometry data analysis (TreeStar, Ashland, OR, USA)

2. BD FACSDiva software; a collection of tools for flow cytometer and application setup, data acquisition, and data analysis (BD Biosciences)

## Procedure

This protocol was adapted from the protocol of PrimeFlow RNA Assay (Invitrogen, Thermo Fisher Scientific). We recommend performing the assay in two days’ time: sections A–D take place on Day 1, and sections F–G take place on Day 2. Section E can be prepared 5 days prior to acquisition. Moreover, we strongly recommend including an HIV-negative control and a probe-signal positive control for each individual experiment. In this protocol, we verified the use of PBMCs from blood donors and reactivated ACH-2 cells as negative and positive assay controls, respectively.

**A. Cell sample preparation**

1. Optional positive control: seed 5 × 10^6^ ACH-2 cells at a density of 1 × 10^6^ cells per mL on Day 0. Stimulate with TNF-α (10 ng/mL) overnight.

2. Prewarm PrimeFlow RNA wash buffer to room temperature. Aliquot volume needed for the day.

3. Fresh isolated PBMCs can be used directly. If cells are frozen, thaw accordingly before use. If using cultured cells, harvest cells from culture flasks or plates. For efficient recovery, use 2–5 × 10^6^ cells per sample.

4. Transfer cells in their respective medium to PrimeFlow RNA 1.5 mL tubes. Centrifuge at a suitable speed and time to pellet cells (e.g., 400× *g* for 5 min). Discard supernatant.

5. Wash with 1 mL of PBS and centrifuge at a suitable speed and time. Discard supernatant.

**B. Surface antibody staining (optional), and fixation**

1. Optional (or skip to step B2): stain cells with fluorescently labeled antibodies for surface markers at optimal concentrations in FACS buffer. Incubate for 30 min in the dark at 4 °C. Cells may also be stained accordingly with a fixable viability dye before or after surface staining.

*Note: We recommend titrating antibodies in advance to determine their optimal concentrations for flow cytometric detection.*

**Critical:** Fluorescent samples should be kept in the dark from this step onward.

2. Wash with 1 mL of FACS buffer. Centrifuge at a suitable speed and time. Discard supernatant.

3. To preserve short abortive HIV-1 transcripts, treatment with PrimeFlow microRNA pretreatment buffer is recommended. Prepare 1 mL of PrimeFlow microRNA pretreatment buffer per sample by diluting 0.25 mL of pretreatment concentrate (4×) with 0.75 mL of pretreatment diluent. Mix gently by inverting. Protect this working solution from light and keep it at room temperature.

4. Add 1 mL of prepared PrimeFlow microRNA pretreatment buffer to each sample and pipette carefully to mix. Incubate for 15 min in the dark at room temperature.

**Caution:** PrimeFlow microRNA pretreatment buffer contains formaldehyde.

5. Centrifuge at 800× *g* for 5 min at 4 °C. Discard supernatant.

**Critical:** Increase centrifugation speed once cells are fixed.

6. Wash with 1 mL of PBS. Centrifuge at 800× *g* for 5 min at 4 °C. Discard supernatant.

7. Prepare 1 mL of PrimeFlow RNA fixation buffer 1 per sample using 0.5 mL of fixation buffer 1A and 0.5 mL of fixation buffer 1B. Mix gently by inverting.

**Caution:** PrimeFlow RNA fixation buffer 1A contains formaldehyde.

8. Fix cells by adding 1 mL of prepared fixation buffer 1 to each sample and invert to mix. Incubate for 30 min in the dark at 4 °C.

9. Centrifuge at 800× *g* for 5 min at 4 °C. Discard supernatant.

**C. Permeabilization, intracellular antibody staining, and intracellular fixation**

1. Prepare 2 mL of PrimeFlow RNA permeabilization buffer with RNase inhibitors per sample by diluting 0.2 mL of PrimeFlow RNA permeabilization buffer (10×) with 1.8 mL of RNase-free water, and add RNase inhibitors at a 1/100 dilution. Mix gently by inverting and keep at 4 °C.

2. Add 1 mL of prepared PrimeFlow RNA permeabilization buffer with RNase inhibitors to each sample, invert to mix, and centrifuge at 800× *g* for 5 min at 4 °C. Discard supernatant.

3. Repeat step C2.

4. Optional (or skip to step C6): Stain cells with fluorescently labeled antibodies detecting intracellular proteins at optimal concentrations in PrimeFlow RNA permeabilization buffer with RNase inhibitors. Incubate for 30 min in the dark at 4 °C.

*Note: We recommend titrating antibodies in advance to determine their optimal concentrations for flow cytometric detection.*

5. Wash with 1 mL of PrimeFlow RNA permeabilization buffer with RNase inhibitors. Centrifuge at 800× *g* for 5 min at 4 °C. Discard supernatant.

6. Prepare 1 mL of PrimeFlow RNA fixation buffer 2 per sample by diluting 0.125 mL of PrimeFlow RNA fixation buffer 2 (8×) with 0.875 mL of PrimeFlow RNA wash buffer. Mix gently by inverting and keep at room temperature.

7. Fix cells by adding 1 mL of prepared PrimeFlow RNA fixation buffer 2 to each sample and invert to mix. Incubate for 1 h in the dark at room temperature.

8. Centrifuge at 800× *g* for 5 min at room temperature. Discard supernatant.

9. Wash twice with 1 mL of PrimeFlow RNA wash buffer at room temperature. After the final wash, aspirate all but 100 μL of supernatant using the markings on the PrimeFlow RNA 1.5 mL tube and resuspend cells in the residual volume.

**D. Target probe hybridization**

1. Thaw target probe sets (20×) at room temperature and prewarm PrimeFlow RNA target probe diluent to 40 °C. Prepare 100 μL of diluted target probes (1×) per sample. Combine target probe sets if using more than one, and adjust the volume of the PrimeFlow RNA target probe diluent accordingly so that the final volume remains 100 μL per sample.

For example, to achieve 1× concentration of target probes, add 5 μL of target probe to 95 μL of diluent. When using two target probe sets, add 5 μL of target probe 1 and 5 μL of target probe 2 in 90 μL of diluent.

**Caution:** PrimeFlow RNA target probe diluent contains formamide.

2. Add 100 μL of diluted target probes (1×) directly into the cell suspension of the appropriate sample and pipette carefully to mix. Then incubate for 2 h in the dark at 40 °C. Invert samples to mix after 1 h.

**Critical:** This step is highly temperature dependent. Please ensure holding temperature at 40 ± 1 °C.

3. Wash twice with 1 mL of PrimeFlow RNA wash buffer at room temperature.

4. Add RNase inhibitors at a 1/100 dilution to PrimeFlow RNA wash buffer.

5. Add 1 mL of PrimeFlow RNA wash buffer with RNase inhibitors to each sample, invert to mix, and centrifuge at 800× *g* for 5 min at room temperature. Aspirate all but 100 μL of supernatant and resuspend cells in the residual volume.

**Pause point:** Store samples overnight in the dark at 4 °C.

**E. Compensation controls for target probes and antibodies**

*Note: Compensation controls can be prepared at any time, maximally 5 days prior to acquisition.*

1. Prepare single-color compensation control samples using the PrimeFlow Compensation kit. Label a tube for each fluorochrome that will be used in the experiment.

2. Mix UltraComp eBeads^TM^ microspheres by pulse-vortexing. Add 1 drop of UltraComp eBeads^TM^ microspheres to each tube.

3. For each fluorochrome of the target probes used, add 5 μL of the appropriate compensation control to the appropriate tube. If antibodies were used, add an equal amount for one test sample. Mix briefly by pulse vortexing. Incubate for 15–30 min in the dark at 4 °C.

4. Wash with 1 mL of FACS buffer and centrifuge at 600× *g* for 5 min at room temperature. Aspirate all but 100 μL of supernatant.

5. Add 100 μL of IC fixation buffer to each tube and briefly vortex to mix.

**Caution:** IC fixation buffer contains formamide.

**Pause point:** Store in the dark at 4 °C for up to 5 days.

**F. Probe signal amplification**

1. Prewarm samples and PrimeFlow RNA wash buffer to room temperature. Prewarm PrimeFlow RNA PreAmp mix to 40 °C.

2. Add 100 μL of PrimeFlow RNA PreAmp mix directly into the cell suspension of each sample and briefly pipette to mix. Incubate for 1.5 h at 40 °C.

**Caution:** PrimeFlow RNA PreAmp mix contains formamide.

3. Prewarm RNA Amp mix at 40 °C.

4. Add 1 mL of PrimeFlow RNA wash buffer to each sample, invert to mix, and spin down at 800× *g* for 5 min at room temperature. Aspirate all but 100 μL of supernatant.

5. Repeat step F4 twice.

6. Add 100 μL of PrimeFlow RNA Amp mix directly into the cell suspension of each sample and briefly pipette to mix. Incubate for 2 h at 40 °C.

7. Add 1 mL of PrimeFlow RNA wash buffer to each sample, invert to mix, and spin down at 800× *g* for 5 min at room temperature. Aspirate all but 100 μL of supernatant.

8. Repeat step F7.

9. Thaw PrimeFlow RNA label probes (100×) on ice in the dark. Prewarm RNA label probe diluent to 40 °C. Dilute 1 μL of PrimeFlow RNA label probes (100×) in 99 μL of PrimeFlow RNA label probe diluent for each sample.

10. Add 100 μL of diluted label probes directly into the cell suspension of each sample and briefly pipette to mix. Then incubate for 1 h at 40 °C.

11. Add 1 mL of PrimeFlow RNA storage buffer or FACS buffer to each sample and invert to mix. Centrifuge at 800× *g* for 5 min at room temperature. Aspirate all but 100 μL of supernatant.

12. Repeat step F11.

13. Transfer samples to FACS tubes and resuspend in an appropriate volume of PrimeFlow RNA storage buffer or FACS buffer for acquisition.

**Optional pause point:** Store samples overnight in the dark at 4 °C for acquisition on the following day.

**G. Sample acquisition**

1. Before acquiring samples on a flow cytometer with FACSDiva software, set up compensation for analysis. Wash prepared compensation controls with FACS buffer and centrifuge at 600× *g* for 5 min at room temperature. Aspirate all but 100 μL of supernatant and resuspend in an appropriate volume of FACS buffer.

2. Acquire single-color stains and an unstained sample for compensation. Adjust voltages to position positive and negative populations within scale and ensure clear separation between negative and positive peaks for each fluorochrome.

3. Use the compensation software to assign each tube to its corresponding fluorochrome, and gate on the bead population to define the positive and negative subsets for each fluorochrome. Calculate the compensation matrix to subtract spectral spillover.

4. Apply the compensation matrix to the experiment during acquisition or in analysis.

5. Set up a gating strategy with forward (FSC) and side scatter (SSC), FSC-H and FSC-W, fluorophores of antibodies used in the staining, and fluorophores of the probes used in the staining. Optional: A dump channel can be included to remove the background signal in downstream analysis.

**Critical:** Verify that the probe staining was successful by measuring the positive control.

6. Acquire samples. Make sure to acquire enough cells for each sample so that the probe signal can be distinguished from noise (e.g., at least 5 × 10^5^–1 × 10^6^ cells for PBMC samples).

## Data analysis

**Figure 1.**
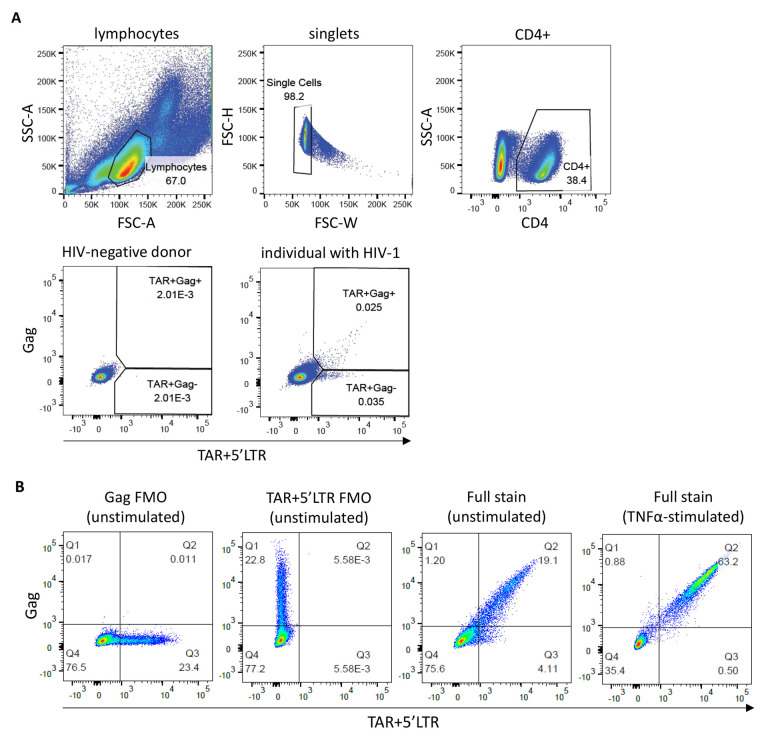
Flow-FISH staining of peripheral blood mononuclear cells (PBMCs) and ACH-2 cells. (A) Gating strategy for PBMCs from a representative HIV-negative blood donor and an individual with HIV-1. Cells were stained with a CD4 antibody and the HIV-1 TAR, 5′LTR, and Gag flow-FISH probes. Gates for the TAR^+^Gag- and double-positive TAR^+^Gag^+^ cell populations are based on the HIV-negative control. (B) FACS plots showing unstimulated ACH-2 cells [fluorescence minus one (FMO) controls and a full stain] and overnight TNF-α-stimulated ACH-2 cells (full stain).

1. Analyze the obtained data using FlowJo. Review and adjust the compensation matrix if needed.

2. Follow the gating strategy described above (Section G; Figure 1) to distinguish the cell populations expressing elongated (TAR^+^Gag^+^) and abortive (TAR^+^Gag-) HIV-1 transcripts. To start, gate out dead cells or cellular debris using FSC and SSC.

**Optional:** Performing cell viability staining can be helpful in assessing live cell populations (step B1).

3. Next, gate on singlets by FSC-H and FSC-W/FSC-A.

4. If surface antibody staining has been performed, e.g., CD4 antibody staining, gate on the cell population of interest before proceeding to probe gating.

**Optional:** Gate out background signal if a dump channel was included during acquisition.

5. Within the cell population of interest (e.g., CD4^+^ lymphocytes), set the antibody parameters on TAR^+^5′LTR-Alexa Fluor 647 and Gag-Alexa Fluor 488. Draw quadrants or gates based on the HIV-negative sample to define TAR^+^Gag-, double-positive TAR^+^Gag^+^, and double-negative TAR-Gag- populations.

**Critical:** It is essential to define population gates independently for each experiment to account for variability in fluorescence. We recommend including an HIV-negative control in each experimental run.

## Validation of protocol

This protocol or parts of it have been used and validated in the following research article:

• Man et al. [10]. Transcriptomic HIV-1 reservoir profiling reveals a role for mitochondrial functionality in HIV-1 latency. *PLOS Pathogens*, 2025. https://doi.org/10.1371/journal.ppat.1012822

Figure 1 of the research article shows unstimulated ACH-2 cells with low-level HIV-1 transcriptional activity, and TNF-α-stimulated ACH-2 cells in which the major population is found to be TAR^+^Gag^+^ after transcriptional induction of provirus by TNF-α. Moreover, a spike-in titration with known frequencies of TFN-α-stimulated ACH-2 cells in uninfected blood donor PBMC is shown (simple linear regression, *r* ^2^ = 0.9996).

## General notes and troubleshooting

**General notes**

1. Up to four different target probes may be used simultaneously. We opted for Alexa Fluor 647 for the TAR and 5′LTR probes, as this probe type provides high sensitivity that is best for low or unknown expression levels of targets.

2. Probes can be designed for other RNA targets of interest (e.g., HIV-1 clades of HIV-2, cellular transcripts).

3. The assay can be used in combination with multiple surface or intracellular antibodies. PerCP, PerCP-Cyanine5.5, and PerCP-eFluor^TM^ 710 should not be used, and it is recommended to avoid tandem dyes, if possible.

4. It is recommended to include single stain and fluorescence minus one (FMO) controls as they help define the true fluorescent signal of the probes.

5. Detected population frequencies in biological samples are widely variable. Our estimates for TAR^+^Gag- and TAR^+^Gag^+^ cell populations for viremic individuals were between 127 and 412 and 103 and 892/10^6^ CD4^+^ T cells, respectively; TAR^+^Gag- and TAR^+^Gag^+^ cell populations for ART-suppressed individuals were between 98 and 436 and 30 and 312/10^6^ CD4^+^ T cells, respectively.

6. This assay can be adapted for the quantification of the HIV-1 reservoir in lymphoid tissues. We suggest processing biopsy materials accordingly and starting this assay with the harvested lymphocytes from these tissues.

7. Before starting major experiments using indispensable biological materials, we recommend assessing batch-to-batch variability of kit reagents and probes using HIV-infected and uninfected cell lines with known or estimated frequencies of probe-positive populations.

**Troubleshooting**

Problem 1: Loss of cells during wash or probe hybridization steps.

Possible cause: Low centrifugation speed and/or short centrifugation time of fixed cells, improper homogenization of the sample and buffers, or suboptimal pipetting and mixing techniques.

Solution: Fixed cells require a higher centrifugal speed; ensure centrifugation is set to the right speed following fixation. Moreover, ensure proper homogenization of the sample and added buffers prior to centrifugation. Hybridization buffer is especially viscous; if necessary, incubate for 1 min after mixing to allow homogenization of the sample. Ensure cell resuspension is performed using the specified mixing techniques described in the protocol. Reduce wash steps if cell loss is high (this may increase the background signal).

Problem 2: Auto-fluorescence or high background noise during acquisition and analysis.

Possible cause: Cells become more autofluorescent following fixation. Some level of background signal is inevitable.

Solution: Noise can be minimized using a dump channel. Acquire as many cells as possible and draw tighter gates to attain the true signal for the respective populations (this may increase the incidence of false negative events).

Problem 3: Low probe signal.

Possible cause: Probes are not hybridized properly.

Solution: Make sure to properly follow the steps of the protocol, as temperature changes may influence the probe hybridization and signal amplification steps.

Problem 4: Low frequencies of probe signal-positive cells detected.

Possible cause: Biological differences in samples containing HIV-1-infected cells (e.g., small HIV-1 reservoir), or cells may not express abortive or elongated HIV-1 transcripts.

Solution: When the HIV-1 reservoir size is expected to be low (e.g., in ART-treated individuals), it is advised to acquire more cells. Moreover, reactivation of cells using various stimuli may be attempted to induce the expression of elongated HIV-1 transcripts. In this case, a critical and nuanced interpretation is essential prior to drawing conclusions regarding the HIV-1 reservoir.

## Supplementary information

The following supporting information can be downloaded here:

1. Table S1. Sequences used for the design of FISH probes
